# Transbronchial biopsy from the upper pulmonary lobes is associated with increased risk of pneumothorax – a retrospective study

**DOI:** 10.1186/s12890-019-0820-z

**Published:** 2019-03-01

**Authors:** Vladimir Herout, Michaela Heroutova, Zdenek Merta, Ivan Cundrle Jr, Kristian Brat

**Affiliations:** 1 Department of Respiratory Diseases, University Hospital Brno and Faculty of Medicine, Masaryk University, Jihlavska 20, 62500 Brno, Czech Republic; 2 Department of Anesthesiology and Intensive Care, St. Anne’s University Hospital and Faculty of Medicine, Masaryk University, Brno, Czech Republic; 3 International Clinical Research Center, St. Anne’s University Hospital, Brno, Czech Republic

**Keywords:** Pneumothorax, Bronchoscopy, Transbronchial biopsy, Upper pulmonary lobe, Pleural pressure

## Abstract

**Background:**

Pneumothorax (PTX) is one of the most common complications of transbronchial biopsy (TBB). Previous research suggests that upper pulmonary lobe TBB may be associated with increased risk of PTX development. The aim of this study was to compare the risk of PTX after TBB performed from different pulmonary lobes.

**Methods:**

All bronchoscopic records from the period January 1st, 2015 - December 31st, 2017 (from the Department of Respiratory Diseases, University Hospital Brno, Czech Republic) were retrospectively analyzed. Of the 3542 bronchoscopic records, 796 patients underwent TBB and were further analyzed. Basic demographic data, TBB procedure-related factors, smoking history and radiological features were analyzed. Furthermore, in patients who developed PTX, PTX onset, PTX symptoms, distribution of the abnormal radiological findings and duration of hospitalization were also analyzed.

**Results:**

Patients who developed PTX had significantly lower body mass index (BMI) and more than 4 samples taken during procedure (all *p* < 0.05). TBB performed from the left upper pulmonary lobe was associated with a significant risk of PTX development (OR 2.27; 95% CI 1.18–4.35; *p* = 0.02). On the contrary, TBB performed from the right lower lobe was associated with a significant reduction of risk of developing PTX (OR 0.47; 95% CI 0.22–0.98; *p* = 0.04). Logistic regression analysis showed BMI (OR 1.08; 95% CI 1.02–1.16; *p* = 0.01), left upper lobe as sampling site (OR 2.15; 95% CI 1.13–4.11; *p* = 0.02) and more than 4 samples taken (OR 1.91; 95% CI 1.04–3.49; *p* = 0.04) to be significantly associated with PTX development.

**Conclusions:**

We conclude that TBB from the left upper pulmonary lobe is associated with significantly increased risk of post-procedural PTX. The right lower pulmonary lobe seems to be the safest sampling site to perform TBB. In patients with diffuse-type pulmonary disease, TBB should be performed preferably from the right lower lobe in order to decrease the risk of post-procedural PTX.

## Background

Transbronchial lung biopsy (TBB) is a procedure performed during flexible bronchoscopy with the use of biopsy forceps. Usually, the purpose is to obtain samples of peripheral lung tissue in order to diagnose interstitial lung disease or to specify the character of a peripheral lung lesion. The first experience with TBB using flexible bronchoscopy was published by Levin et al. in 1974 [1].

The most common complications of TBB are pneumothorax (PTX) and bleeding. An overall complication rate was reported to be around 6% [2]. The incidence of PTX after TBB was reported to be 1–6% [3]. The main risk factors include emphysema, COPD and positive pressure ventilation [4, 5]. In a study by Huang et al., TBB performed from the upper pulmonary lobes tended to be a potential risk factor for PTX development when compared to other locations [4]. However, in this study, the group of patients with PTX was too small to gain statistical significance [4, 5].

An outpatient bronchoscopy procedure is usually performed in a supine position. However, if no complications occur within 2 h from the procedure, the patient is allowed to leave the health-care facility (in an upright position). In a healthy human in an upright position, the pleural pressure is most negative in the apical parts and least negative in the basal parts of the pleural space [6]. A second point is that the alveoli in the upper parts of the lung are larger than those in the lower parts. This is the consequence of the apico-basal pressure gradient in the pleural cavity resulting in a higher surface tension of the apical lung tissue [7]. It is also a possible reason for the subpleural blebs formation in the apex of the lungs [6]. The existence of the apico-basal gradient in pleural pressure may favour the development post-procedural PTX after TBB from the upper pulmonary lobes [6]. We hypothesized that the incidence of PTX will be higher in patients after TBB performed from the upper than from lower pulmonary lobes. Accordingly, the aim of this study was to evaluate the incidence of PTX in TBB performed from diffent lung lobes.

## Methods

### Background

Department of Respiratory Diseases, University Hospital Brno, Czech Republic, is a university-type facility concentrating the most difficult respiratory cases (i.e. patients with lung cancer, interstitial lung disease, cystic fibrosis etc.) from an area populated by ca 1.5 million inhabitants. Annually, cca. 1100–1300 bronchoscopic procedures are performed in the institution. Of these, cca. 250–300 patients annually undergo TBB. The most frequent indication for TBB include suspicion of lung cancer, differential diagnosis of interstitial lung disease and infiltrates of unknown significance present on chest radiograph or lung CT scan.

Flexible fiberoptic bronchoscopies were performed in light sedation (using 0.25 mg of alprazolam or 3.75 mg of midazolam) and using topical anesthetics (lidocaine and trimecaine or bupicavaine) in accordance with the 2013 British Thoracic Society (BTS) guidelines [3]. Flexible fiberoptic bronchoscopes type BF-1 T180 (Olympus, Japan) and EB-1975 K (Pentax Medical, Japan) were used. Fluoroscopically unguided TBB were performed in accordance with the BTS guidelines [3] using the 1.8 mm-diameter Single Use-Biopsy Forceps - type NBF12–11018120 (Micro-Tech, China).

### Study population

All bronchoscopic records from the period between January 1st, 2015 and December 31st, 2017 were retrospectivelly analyzed. Patients with TBB were included in the study. From the patient’s records, demographic data (age, weight, height, BMI, gender), PTX incidence, indication for TBB, number of samples taken during TBB, area of TBB sampling (pulmonary lobe), smoking history and CT or chest X-ray (CXR) signs of emphysema were analyzed. Furthermore, in patients who developed PTX, onset of PTX (< 24 h, > 24 h), PTX symptoms (cough, chest pain, desaturation > 5%, dyspnoea or none), PTX treatment (conservative treatment, desuflation, chest tube insertion), hospital length of stay (LOS) and mortality were analyzed.

### Statistical methods

The Shapiro-Wilk test was used to evaluate normality. Comparisons between patients with and without PTX were made by the Student t-test and Mann-Whitney U test. Differences in proportions were tested by the 2 × 2 table and two-tailed Fisher exact test. Multivariate stepwise logistic regression was used to analyze which of the parameters was independently associated with the development of PTX. Parameters that were significantly different between both groups (with PTX vs. without PTX) and had OR > 1.00 were included in the logistic regression model (BMI, number of samples taken, sampling site). Data are summarized as mean ± standard deviation (SD); *p* values < 0.05 were considered statistically significant. Statistica software 12.0 (StatSoft Inc., Prague, Czech Republic) was used for statistical analysis.

## Results

A total of 3542 bronchoscopic records were analyzed. Seven hundred and ninety-six patients underwent the TBB procedure. Out of the analyzed 796 patients, 49 patients (6.16%) developed PTX. Basic demographic data and procedure-related characteristics are presented in Table 1. Of the 796 patients, 58% were men, 55% were current or past smokers, mean age was 63 years. The most frequent indication for TBB was a nodular lesion (48%) followed by diffuse lung disease (24%); an upper lobe was used as sampling site in 33%, middle lobe in 15% and a lower lobe in 46% of the cohort. Six percent of patients underwent TBB from more than one lobe. A comparison between patient groups with and without PTX is shown in Table 2. Patients who developed PTX had significantly lower BMI, more than 4 samples taken during procedure. Furthermore, TBB was more frequently performed from the left upper lobe in subjects with PTX. On the contrary, the right lower lobe was more frequently used as a site for TBB in subjects without PTX. In patients with diffuse-type lung pathology (*n* = 187), the diagnostic yield of TBB was 52.9% in our cohort. In patients with nodular lesions, the diagnostic yield of the TBB procedure was 40.5% in our cohort.

**Table 1:** Basic characteristics of the study cohort (*n* = 796)

Age (years)	63 ± 13
Male No. (%)	461 (58)
BMI (kg/m^2^)	28 ± 14
No of samples ≥4 No. (%)	199 (25)
PTX No. (%)	49 (6)
Indication to TBB	
Nodular lesion (suspicion of tumour) No. (%)	385 (48)
Diffuse lung disease (suspicion of pulmonary fibrosis) No. (%)	187 (24)
Infiltrates of unknown etiology No. (%)	165 (21)
Other No. (%)	59 (7)
Sampling location (pulmonary lobe)	
Sample from RUL No. (%)	136 (17)
Sample from RML No. (%)	122 (15)
Sample from RLL No. (%)	252 (32)
Sample from LUL No. (%)	126 (16)
Sample from LLL No. (%)	114 (14)
Sample from > 2 lobes No. (%)	46 (6)
Emphysema on lung CT or CXR No. (%)	218 (27)
Smoking No. (%)	443 (55)

*BMI* body mass index, *CT* computed tomography, *CXR* chest radiograph, *kg* kilogram, *LLL* left lower lobe, *LUL* left upper lobe, *m* meter, *No*. number, *RLL* right lower lobe, *RML* right middle lobe, *RUL* right upper lobe

**Table 2:** Comparison of patients with and without PTX

Parameter	without PTX (*n* = 747)	with PTX (*n* = 49)	OR	95% CI	p
Age (years)	63 ± 13	66 ± 11	0.98	0.95–1.00	0.06
Male No. (%)	430 (58)	31 (63)	1.27	0.70–2.31	0.46
BMI (kg/m^2^)	28 ± 15	25 ± 5	1.09	1.02–1.16	0.01
No. of samples ≥4 No. (%)	180 (24)	19 (39)	2.00	1.10–3.63	0.03
Indication to TBB					
Nodular lesion (suspicion of tumour) No. (%)	355 (47)	30 (61)	1.74	0.96–3.15	0.08
Diffuse lung disease (suspicion of pulmonary fibrosis) No. (%)	173 (23)	14 (29)	1.33	0.70–2.52	0.49
Infiltrates of unknown etiology No. (%)	162 (22)	3 (6)	0.24	0.07–0.77	< 0.01
Other No. (%)	57 (8)	2 (4)	0.52	0.12–2.18	0.42
Sampling location (pulmonary lobe)					
Sample from RUL No. (%)	124 (17)	12 (25)	1.63	0.83–3.21	0.17
Sample from RML No. (%)	116 (15)	6 (12)	0.76	0.32–1.82	0.56
Sample from RLL No. (%)	243 (33)	9 (18)	0.47	0.22–0.98	0.04
Sample from LUL No. (%)	112 (15)	14 (29)	2.27	1.18–4.35	0.02
Sample from LLL No. (%)	109 (14)	5 (10)	0.67	0.26–1.71	0.42
Sample from > 2 lobes No. (%)	43 (6)	3 (6)	1.07	0.32–3.57	1.00
Emphysema No. (%)	199 (27)	19 (39)	1.74	0.96–3.17	0.07
Smoking No. (%)	412 (55)	31 (63)	1.40	0.77–2.55	0.30

*BMI* body mass index, *kg* kilogram, *LLL* left lower lobe, *LUL* left upper lobe, *m* meter, *No*. number, *OR* Odds Ratio, *RLL* right lower lobe, *RML* right middle lobe, *RUL* right upper lobe; *95% CI* 95% Confidence Interval

A stepwise logistic regression (BMI, number of samples taken and sampling site were used in the model) is shown in Table 3. All 3 included variables were significantly associated with PTX development; LUL sampling site had the highest OR (OR 2.15; 95% CI 1.13–4.11; *p* = 0.02).

**Table 3:** Logistic regression

parameter	OR	95% CI	p
BMI (kg/m2)	1.08	1.02–1.16	0.01
No. of samples ≥4	1.91	1.04–3.49	0.04
Sample from LUL	2.15	1.13–4.11	0.02

*BMI* body mass index, *kg* kilogram, *LUL* left upper lobe, *m* meter, *No*. number

Of the 49 PTX cases, 31 patients (63%) had early onset of PTX while 18 cases (37%) were diagnosed > 24 h after the procedure. In twenty-seven of the 49 PTX patients (55%), PTX was clinically significant and warranted chest tube insertion. Hospital LOS was significantly longer in patients with thoracic drainage when compared to patients treated conservatively (7 ± 4 vs. 2 ± 1 vs. days; *p* < 0.01). Of the 27 cases of PTX treated by thoracic drainage, 11 patients (41%) had undergone left upper lobe biopsy and 4 of these 11 patients (36%) had pathological lesions present also in other pulmonary lobes. Choosing a different sampling site for TBB in these 4 patients might have decreased the number of clinically significant PTX (i.e. requiring chest tube insertion) by about 15% in our cohort.

## Discussion

The major finding of this study was that the left upper pulmonary lobe TBB, BMI and more than 4 samples taken were significantly associated with increased odds of post-procedural PTX. In contrast, the odds of post-procedural PTX were the lowest with TBB done from the right lower lobe.

In our study, the incidence of PTX was 6.16% which is comparable to the data from the BTS guideline (PTX incidence reported to be 1–6%) [3], the Sindhwani et al. study (10.2%) [8], from the COMET Trial (7.2%) [9] and from the Ibrahim et al. study (9.8%) [10]. Our cohort included 48% of patients with nodular lesions and there was an insignificant trend towards more nodular lesions TBB in the PTX group. Nodular lesions are strongly associated with the presence of pulmonary emphysema and vice versa [11], which may increase the risk of postprocedural PTX development [9, 12]. Indeed, compared to patients without PTX, in patients with PTX there was also an insignificant trend towards more pulmonary emphysema in our cohort. The ideal number of TBB samples that should be taken at each procedure remains controversial, some authors concluded that it has no relevance to risk of post-procedural PTX [9]. In our analysis, the number of samples taken during TBB was significantly associated with PTX development and remained a significant factor of PTX development also in the logistic regression analysis.

In our study, the odds of PTX development were the highest in patients with TBB performed from the left upper lobe. In contrast, the odds were the lowest in patients with TBB performed from the right lower lobe. The observed apico-basal difference may be explained by the vertical pleural pressure gradient. In a healthy human in an upright position, the pleural pressure is the lowest (i.e. most negative) in the apical parts and the highest (i.e. least negative) in the basal parts. The pressure difference between the pulmonary apex (about -12 cm H_2_O) and the base (about -2 cm H_2_O) may be up to 8–10 cmH_2_O [6]. Therefore, a more negative pressure in the apical parts may predispose to the easier PTX development in patients undergoing TBB from the upper pulmonary lobes. Interestingly, there also appears to be a difference in pleural pressure between the right and the left lung. The right lung is heavier and the pleural pressure seems to be higher in the basal part of the right lung when compared to the basal parts of the left lung [13]. This side-difference may explain the right lower lobe TBB association with the lowest odds of PTX development.

The higher incidence of PTX in subjects with TBB from the upper lobe is in concensus with the study of Huang et al. and Fernandez-Bussy et al. [12, 14]. In a study by Huang et al., 13 cases of post-procedural PTX out of 399 procedures were reported after endobronchial ultrasound-guided TBB [12]. In this study, univariate analysis revealed that pulmonary emphysema was associated with increased risk of PTX. Importantly, of the 13 PTX cases, 10 events occured after TBB from the upper lobes and only 3 from the middle and lower lobes. However, this clear trend was statistically insignificant (*p* = 0.084), probably due to the low number of PTX cases in the cohort. In the same study, the multivariate analysis showed that pulmonary emphysema was the strongest independent risk factor of post-TBB PTX with OR 55.09 (*p* < 0.001) while a location of the lesion in the upper lobes had an OR 3.34 albeit insignificant (*p* = 0.149) due to small number of PTX cases [12].

In the study by Fernandez-Bussy et al., upper or middle lobe procedure was found to be an independent risk factor of post-procedural complications (OR 1.69) [14]. This did not reach statistical significance as the study was severely underpowered; only 3 cases of PTX were reported [14].

In contrast to our study, Izbicki et al. reported 8 cases of post-procedural PTX, of which none resulted from an upper pulmonary lobe TBB [15]. However, in most of the studies, the location of TBB sampling was not evaluated in relation to PTX development [2, 9, 12, 16].

The diagnostic yield of TBB (with no fluoroscopy guidance) in our study cohort was 52.9% for diffuse parenchymal lung diseases and 40.5% for nodular lesions, respectively. This is comparable to previous reports where: Hernandez Borge et al. reported an overall diagnostic yield of 42% [17], in a Swiss study (Descombes et al.), the diagnostic yield for diffuse-type lung pathologies was 50% [18]. In contrast, the BTS guideline cited 2 studies where the sensitivity was up to 67–74% [3]. For peripheral lung lesions, a very wide variation rate accross different studies (16–80%) has been reported [19]. In the BTS guideline, diagnostic accuracy of TBB for malignant lesions was around 45%, while in the Swiss study (Descombes et al.) only around 29% [18].

Our study had several limitations. First, it was a retrospective study. Therefore, commenting on causality between the site of TBB and PTX development is difficult. Second, in our cohort, there was an insignificant trend towards more nodular lesion TBBs in the PTX group. Therefore, several TBBs in our cohort could not have been performed from lower lobes in order to prevent PTX development. We suggest that in case the TBB could not be performed from the lower lobes it should at least increase the awareness of PTX development.

Third, all procedures included in the current study performed transbronchial biopsy without fluoroscopy. It is unknown if these results apply to centers that use fluoroscopy as part of their standard practice, however, there is no evidence that fluoroscopy changes the incidence or location of pneumothorax during TBB [3, 15, 20–22].

Last, this was a retrospective study and we were not able to measure the distance of nodules from visceral pleura in all patients with nodular lesions, as some CT scans were no longer available for review. In patients with peripheral lesions, the risk of PTX development may be higher than in patients with more centrally situated lesions. However, we believe the majority of patients with peripheral lesions underwent a CT-guided transthoracic needle biopsy as recomended [23] leaving mostly those patients with more centrally situated lesions in our study cohort. Indeed, in patients with a nodular lesion and with PTX (all the 30 patients had CT scans still available for review), the mean lesion distance from the visceral pleura was 31 mm vs 29 mm for non-PTX patients (*p* = 0.08).

## Conclusions

We conclude that TBB from the left upper pulmonary lobe is associated with significantly increased risk of post-procedural PTX development. In contrast, right lower pulmonary lobe appears to be the safest sampling site to perform TBB.

In patients with a solitary nodular lesion, TBB should be performed under fluoroscopy guidance (if available). The use of fluoroscopy increases the diagnostic yield and may decrease the risk of procedure-related PTX [3].

In patients with a diffuse-type (parenchymal) lung disease, the use of fluoroscopy does not improve the diagnostic yield of TBB and is not routinely recommended. Our data show that the risk of post-TBB PTX may be decreased by choosing the lower lobes (preferably RLL) as sampling site instead of an upper-lobe procedure.

In patients with nodular lesions in multiple locations (e.g. multiple metastases) and where fluoroscopy is not available, the lower lobes should be chosen preferably as a sampling site.

A prospective study addressing the impact of TBB sampling site on risk of postprocedural PTX in a more strictly defined study population (e.g. solely diffuse lung disease patients or solely patients with nodular lesions) with and without fluoroscopy guidance would be beneficial.

## Abbreviations

BMI: Body Mass Index
CT: Computed Tomography
LOS: Length of stay
PTX: Pneumothorax
TBB: Transbronchial biopsy
